# Onset features and time to diagnosis in Friedreich’s Ataxia

**DOI:** 10.1186/s13023-020-01475-9

**Published:** 2020-08-03

**Authors:** Elisabetta Indelicato, Wolfgang Nachbauer, Andreas Eigentler, Matthias Amprosi, Raffaella Matteucci Gothe, Paola Giunti, Caterina Mariotti, Javier Arpa, Alexandra Durr, Thomas Klopstock, Ludger Schöls, Ilaria Giordano, Katrin Bürk, Massimo Pandolfo, Claire Didszdun, Jörg B. Schulz, Sylvia Boesch, on behalf of the EFACTS (European Friedreich’s Ataxia Consortium for Translational Studies)

**Affiliations:** 1 Department of Neurology, Medical University of Innsbruck, Anichstrasse 35, 6020 Innsbruck, Austria; 2 Department of Public Health, Health Services Research and Health Technology Assessment, UMIT - University of Health Sciences, Medical Informatics and Technology, Hall in Tirol, Austria; 3 Department of Molecular Neuroscience, UCL Institute of Neurology, London, UK; 4 Unit of Genetics of Neurodegenerative and Metabolic Diseases, Fondazione IRCCS Istituto Neurologico Carlo Besta, Milan, Italy; 5 Reference Unit of Hereditary Ataxias and Paraplegias, Department of Neurology, IdiPAZ, Hospital Universitario La Paz, Madrid, Spain; 6 Sorbonne Université, Institut du Cerveau et de la Moelle épinière (ICM), AP-HP, Inserm U 1127, CNRS UMR 7225, University Hospital Pitié-Salpêtrière, Paris, France; 7 Department of Neurology with Friedrich-Baur-Institute, University of Munich, Munich, Germany; 8 German Center for Neurodegenerative Diseases (DZNE), Munich, Germany; 9 Department of Neurodegenerative Diseases, Hertie-Institute for Clinical Brain Research, University of Tübingen, Tübingen, Germany; 10 German Center for Neurodegenerative Diseases (DZNE), Tübingen, Germany; 11 Department of Neurology, University Hospital of Bonn, Bonn, Germany; 12 German Center for Neurodegenerative Diseases (DZNE), Bonn, Germany; 13 Department of Neurology, Philipps University of Marburg, Marburg, Germany; 14 Laboratory of Experimental Neurology, Université Libre de Bruxelles, Brussels, Belgium; 15 Department of Neurology, RWTH Aachen University, Aachen, Germany; 16 JARA-BRAIN Institute of Molecular Neuroscience and Neuroimaging, Forschungszentrum Jülich GmbH and RWTH Aachen University, Aachen, Germany

**Keywords:** Friedreich’s Ataxia, Age at onset, Genetic testing, Diagnostic delay, Natural history study

## Abstract

**Background:**

In rare disorders diagnosis may be delayed due to limited awareness and unspecific presenting symptoms. Herein, we address the issue of diagnostic delay in Friedreich’s Ataxia (FRDA), a genetic disorder usually caused by homozygous GAA-repeat expansions.

**Methods:**

Six hundred eleven genetically confirmed FRDA patients were recruited within a multicentric natural history study conducted by the EFACTS (European FRDA Consortium for Translational Studies, ClinicalTrials.gov-Identifier NCT02069509). Age at first symptoms as well as age at first suspicion of FRDA by a physician were collected retrospectively at the baseline visit.

**Results:**

In 554 of cases (90.7%), disease presented with gait or coordination disturbances. In the others (*n* = 57, 9.3%), non-neurological features such as scoliosis or cardiomyopathy predated ataxia. Before the discovery of the causal mutation in 1996, median time to diagnosis was 4(IQR = 2–9) years and it improved significantly after the introduction of genetic testing (2(IQR = 1–5) years, *p* < 0.001). Still, after 1996, time to diagnosis was longer in patients with a) non-neurological presentation (mean 6.7, 95%CI [5.5,7.9] vs 4.5, [4.2,5] years in those with neurological presentation, *p* = 0.001) as well as in b) patients with late-onset (3(IQR = 1–7) vs 2(IQR = 1–5) years compared to typical onset < 25 years of age, *p* = 0.03).

Age at onset significantly correlated with the length of the shorter GAA repeat (GAA1) in case of neurological onset (r = − 0,6; *p* < 0,0001), but not in patients with non-neurological presentation (r = − 0,1; *p* = 0,4). Across 54 siblings’ pairs, differences in age at onset did not correlate with differences in GAA-repeat length (r = − 0,14, *p* = 0,3).

**Conclusions:**

In the genetic era, presentation with non-neurological features or in the adulthood still leads to a significant diagnostic delay in FRDA. Well-known correlations between GAA1 repeat length and disease milestones are not valid in case of atypical presentations or positive family history.

## Introduction

Friedreich ataxia (FRDA) is a rare inherited movement disorder with a prevalence of about 1/50000 in Caucasians [1]. FRDA usually presents around puberty with slowly progressive instability, dysmetria and dysarthria, leading to loss of independent gait and severe disabilities [2]. Ataxia is primarily afferent and is accompanied by sensory loss and abnormalities of deep tendon reflexes [3–5]. The full-blown phenotype of FRDA is characterized also by prominent non-neurological manifestations including cardiomyopathy, diabetes mellitus and skeletal deformities such as scoliosis and pes cavus [2, 6, 7]. In 96% of cases, FRDA is underlain by homozygous GAA triplet expansions in the first intron of the frataxin gene (*FXN*) [8]. Approximately 4% of patients bear one GAA expansion and a different defect such as a point mutation or a small deletion [8, 9]. The length of the expanded repeats, and particularly the length of the shorter one (GAA1), inversely correlates with age at disease onset, disease severity and rate of progression [6, 10, 11].

Rare diseases represent a challenge for medical management, starting from the initial disease recognition. In 2004, a European survey on a subset of rare diseases reported that in 25% of cases diagnosis is achieved between 5 and 30 years after presentation [12]. In a more recent report from the global commission for rare diseases, average time to diagnosis in orphan disorders was estimated to be about 5 years [13]. Limited awareness and non-specific onset symptoms may lead to a series of referrals, prescriptions of redundant tests and ultimately to a diagnostic delay.

To the best of our knowledge, no systematic study addressed the issue of diagnostic delay in FRDA. In our experience, patients’ referral to the specialist is usually prompted by onset of progressive balance and coordination disturbances [2, 14]. Child and adult neurologists then suspect FRDA based on the usually typical constellation of slowly progressive ataxia, areflexia and sensory loss in young patients with healthy parents. However, exceptions to this paradigm do exist. Genetic testing for FRDA may be prompted by heart disease [15, 16]. In two cases of heart disease as the inaugural symptom, FRDA diagnosis was achieved post-mortem, after sudden death [15], or during follow-up after cardiac transplantation [16]. Furthermore, since the discovery of *FXN* mutations as the cause of FRDA in 1996 and the widespread practice of GAA repeat testing, late-onset cases (≥25 years old) due to smaller GAA expansions are increasingly recognized [17, 18]. Late-onset cases are often associated with atypical features such as preserved deep tendon reflexes or even hyperreflexia with spasticity [18–20]. Skeletal deformities and cardiomyopathy are far less prevalent when the disease starts in adulthood [6, 10].

Herein, we addressed the issue of diagnostic delay in FRDA. Particularly, we investigated 1) the impact of the introduction of genetic testing in the diagnostic work-up as well as 2) the correlation between presenting phenotypes and time to diagnosis. To this purpose, we analyzed onset features in a cohort of 611 individuals with genetically confirmed FRDA recruited within a Europe-wide natural history study conducted by the EFACTS (European Friedreich’s Ataxia Consortium for Translational Studies) [10, 11]. Our aim was to raise awareness about the different clinical presentations of FRDA in order to accelerate the referral to specialized centers and therefore the access to clinical trials when the disability status is still low, as well as to effective therapies in the future.

## Methods

Detailed description of study design and recruitment within the EFACTS project has been reported elsewhere [10]. The EFACTS registry is a prospective study which collects clinical data from genetically confirmed FRDA patients in yearly intervals (ClinicalTrials.gov identifier NCT02069509). The study was approved by the local Human Research Ethics committee of each participating center. Written informed consent was given from each subject before inclusion in the study. Patients recruited from September 2010 till July 2015 were considered for the present analysis. Both patients with homozygous GAA-repeat expansions and compound heterozygote patients bearing one expansion and another mutation on the second allele were included. Basic clinical data of this cohort has been already published elsewhere [7, 11].

At the time of their first EFACTS visit, patients, as well as parents or legal guardian in the case of children, are requested to recall 1) age at onset and 2) symptoms at onset. Inaugural symptoms are collected according to a predefined list and multiple entries can be selected (see Table 1). For the purpose of this study, the categories 1) neurological and 2) non-neurological onset were defined as shown in Table 1. Depending on the age at onset, we also defined the following subgroups: typical-onset (< 25 years old) and late-onset (≥25 years old). This latter distinction is based on evidence of different GAA-repeats lengths, clinical characteristics and progression rate [11].

**Table 1:** Definition of the categories neurological and non-neurological onset based on EFACTS registry entries

Predefined onset symptoms in EFACTS registry	Category Neurological Onset	Category Non-neurological Onset
Instability (yes/no)	At least one of the followings: - Instability - Falls - Others (when a neurological symptom is reported, e.g. clumsiness^a^)	1) None of the followings: - Instability - Falls AND 2) Presence of at least one of the followings: - Scoliosis - Cardiomyopathy - Diabetes mellitus - Others (when a non-neurological symptom is reported, e.g. pes cavus^b^)
Falls (yes/no)		
Scoliosis (yes/no)		
Cardiomyopathy (yes/no)		
Diabetes Mellitus (yes/no)		
Others (yes/no. If yes, symptoms specified in free text)		

^a^Further neurological symptoms are specified in Table 2

^b^All patients with “others” as non-neurological onset symptoms reported pes cavus, apart from one (urinary urgency as presenting symptom)

Based on medical history, age at first suspicion of FRDA by a physician is also collected at the first visit. We considered this moment as diagnosis time-point and not that of the genetic confirmation, since genetic testing 1) was available only after 1996 and 2) in the clinical routine it can be postponed for many reasons other than delayed recognition. The timespan between age at onset and age at suspicion of FRDA reflects the diagnostic delay and is expressed as continuous variable “time to diagnosis” in the present study.

### Statistical analysis

SPSS version 25 was used to perform statistical analysis. Statistical significance was set at *p* < 0.05. Data are reported as percentages, median (interquartile range) or mean (SD or 95%CI) depending on their measure level (whether categorical or continuous) and distribution. Normality was tested by means of Kolmogorov-Smirnov and Shapiro-Wilk tests. The variable time to diagnosis was non-normally distributed over all subgroups and non-normal distribution persisted after both logarithmic and Box-Cox automated data transformation. Subgroups comparison of time to diagnosis was conducted both by means of a) Mann-Whitney U test and of b) ANCOVA to be able to consider covariates [21]. In ANCOVA, bias-corrected and accelerated bootstrap with 1000 iterations was applied to account for non-normality and 95% confidence intervals were reported. Relevant covariates were selected by univariate Mann-Whitney U and Chi squared tests. Any variable having a significant univariate test at level *p* < 0.20 was selected as a candidate multivariate for the analysis of covariance. Correlations between GAA1-repeat length and age at onset were assessed with Spearman’s rho coefficient.

## Results

### Time to diagnosis in FRDA in the genetic era

Data from 619 patients were available. We excluded eight cases due to FRDA diagnosis before symptom onset and thus considered 611 cases for the main analysis (see also Table 3). Twenty patients (3.3%) were compound heterozygous.

Forty-seven percent of patients were female. The median age at examination was 31(IQR = 22–43) years old and the median age at onset of disease was 13(IQR = 9–19) years.

**Fig. 1:**
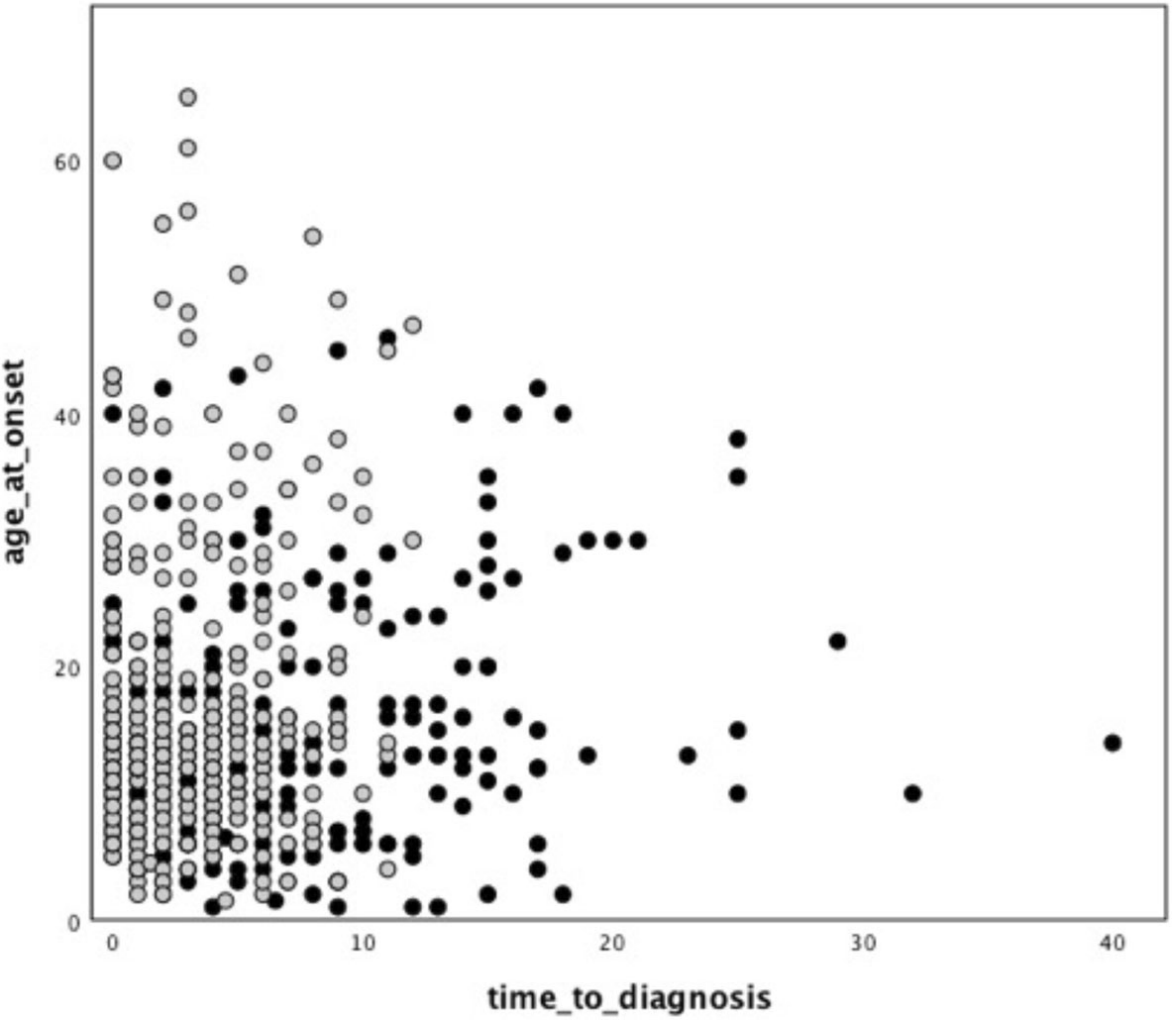
Relationship between age at onset and time to diagnosis, before and after 1996. Time to diagnosis (years) is plotted against age at onset (years). Cases with onset before 1996 are represented by black dots, while cases with onset *after* 1996 are represented by grey dots. A shortening in time to diagnosis after 1996 is evident as well as the presence of several very late onset cases (≥50 years old) detected only after 1996

Time to diagnosis was variable with a median of 3 years, but markedly skewed to the right (IQR = 1–7 years). In 53% of patients disease onset predated the discovery of *FXN* mutation in 1996. Onset before 1996 was associated with a significantly longer time to diagnosis (4(IQR = 2–9) vs 2(IQR = 1–5) years in the cases diagnosed after 1996, *p* < 0.001, see also Fig. 1).

### Neurological versus non-neurological onset

In 90.7% of cases (*n* = 554), disease presented with neurological symptoms. Gait instability was the presenting symptom in the vast majority (*n* = 491, 89%). The patients who did not report gait instability at disease onset described as presenting neurological symptoms clumsiness (*n* = 20, 3.6%), falls (*n* = 19, 3.4%) or problems with hand skills (altogether *n* = 13, 23%). Rarely, patients experienced as first symptoms sensory loss (*n* = 3, 0.5%) or dysarthria (*n* = 2, 0.4%) (see also Table 2).

**Table 2:** Neurological symptoms at onset other than gait instability

Symptom	n. cases (%)
Clumsiness	20 (3.6%)
Falls	19 (3.4%)
Problems with manual dexterity	6 (1.1%)
Difficulties in writing	5 (0.9%)
Hand tremor	3 (0.5%)
Sensory loss in legs	3 (0.5%)
Leg Pain/Stiffness	2 (0.4%)
Slurred Speech	2 (0.4%)
Unspecified neurological symptoms	2 (0.4%)
Asthenia	1 (0.2%)

**Fig. 2:**
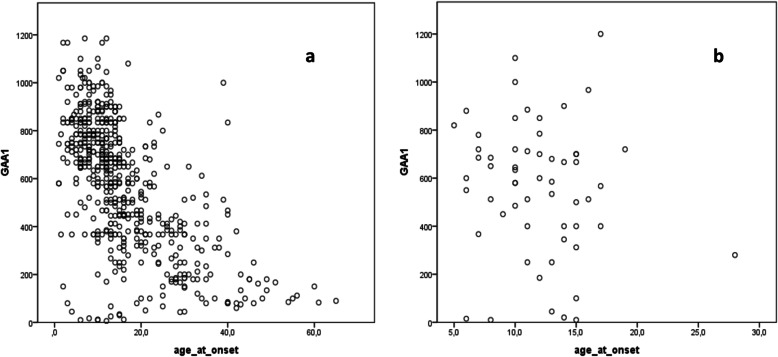
Relationship between age at onset and GAA1-repeat length depending on onset symptoms. The length of the shorter GAA repeat (GAA1) is plotted against age at onset (years) in the groups with (**a**) neurological onset and (**b**) non-neurological onset. As evident in the graphics, a significant correlation between the GAA1 repeat length and age at onset is present in case of classical onset with neurological symptoms (r = − 0,6; *p* < 0,0001), while no correlation is found in the group with non-neurological onset (r = − 0,1; *p* = 0,4)

In 9.3% of cases (*n* = 57) non-neurological symptoms predated ataxia. Scoliosis was the most common inaugural feature in this group (*n* = 48, 84%), followed by cardiomyopathy (*n* = 10, 18%). GAA1-repeat length did not significantly differ between the groups with neurological and non-neurological onset (see Table 3). In the neurological onset group, a strong inverse correlation was found between GAA1-repeat length and age at onset (r = − 0,6; *p* < 0,0001) similarly to previous studies [6, 10, 11]. There was no such significant correlation in the group with non-neurological onset (r = − 0,1; *p* = 0,4, see also Fig. 2). Time to diagnosis in the non-neurological onset subgroup was significantly longer compared to the one with neurological inaugural symptoms (5(IQR = 2–9) vs 3(IQR = 1–6), U = 12,816, *p* = 0.02).

**Table 3:** Clinical features and time to diagnosis in the entire cohort and in the subgroups neurological vs non-neurological onset

	Whole cohort	Neurological onset	Non-Neurological onset	***p*** value
N (%)	611	554 (90,7%)	57 (9,3%)	
Sex (women, %)	286 (47%)	259 (47%)	27 (47%)	0.96
Age at examination (years)	31 (22;43)	32 (23;43)	28 (20;38)	**0.02**
GAA1 (repeat number)	620 (367;785)	634 (367;785)	585 (400;716)	0.43
GAA2 (repeat number)	912 (780;1050)	912 (785;1050)	890 (745;1050)	0.44
Age at onset (years)	13 (9;19)	13 (9;20)	12 (10;15)	**0.03**
Late-onset (≥25 yo) (n. of cases)	103 (17%)	102 (18.4%)	1 (1.7%)	**0.001**
Time to diagnosis (years)	3 (1;7)	3 (1;6)	5 (2;9)	**0.02**

Results of comparisons between the groups neurological vs non-neurological onset are reported in the last column. Categorical and continuous variables were compared by means of Chi-squared test und Mann Whitney-U test respectively. Statistically significant results are reported in bold.

Diagnostic delay in the non-neurological onset subgroup remained significant after controlling for the effect of age at examination, age at onset and presentation before/after 1996 (mean 6.7, 95% CI [5.5, 7.9] vs 4.5, [4.2, 5] years in the neurological onset group, *p* = 0.001, see also Table 4). Since the majority of patients with presentation in adulthood will never develop non-neurological comorbidities (see Table 3 as well as [6, 10, 18]), we repeated the previous analysis considering only typical-onset patients (*n* = 508, of whom 56(11%) presented with non-neurological onset). In the reanalysis, the diagnostic delay due to atypical non-neurological presentation became more evident (mean 6.5, 95% CI [5.2, 7.8] vs 4.1, [3.7, 4.6] years, *p* = 0.0002).

**Table 4:** Comparison of time to diagnosis in neurological vs non-neurological onset by means of ANCOVA

	Mean Time to diagnosis	Standard Error	95% CI	*p* value
*All cohort (n = 611)*				
Neurological onset (n = 554)	4.5	0.197	4.2–5	**0.001**
Non-neurological onset (n = 57)	6.7	0.618	5.5–7.9	
*Typical-onset patients (n = 508)*				
Neurological onset (*n* = 454)	4.1	0.209	3.7–4.6	**0.0002**
Non-neurological onset (*n* = 56)	6.5	0.594	5.2–7.8	

Comparisons were performed both 1) in the entire cohort and 2) in the typical-onset patients. Mean, Standard Error, 95% confidence intervals (CI) and *p* values estimated by bias-corrected and accelerated bootstrap are reported. Estimates are adjusted for age at examination, age at onset and presentation before/after 1996. Statistically significant results are reported in bold.

### Typical- versus late-onset FRDA

In 103 patients (17%) FRDA had a late onset. The time to diagnosis was significantly longer in late-onset FRDA compared to typical-onset FRDA (5(IQR = 2–10) vs 3(IQR = 1–6) years, U = 20,036, *p* < 0.0002). After stratifying for onset before/after 1996, the difference in time to diagnosis remained significant, although was less marked when considering presentations after 1996 (3(IQR = 1–7) vs 2(IQR = 1–5) years U = 8067, *p* = 0.03). In seven cases, FRDA started extremely late (≥50 years old). All of these individuals had presentation after 1996.

### Time to diagnosis in the setting of a positive family history

In the database, 54 siblings’ pairs were identified. As expected, positive family history led to a markedly faster suspicion of FRDA in the subjects who already had an affected sibling (median time to diagnosis 4(IQR = 1–9) vs 1(IQR = 0–4) years, *p* < 0.001 in the comparison between first and second siblings).

**Fig. 3:**
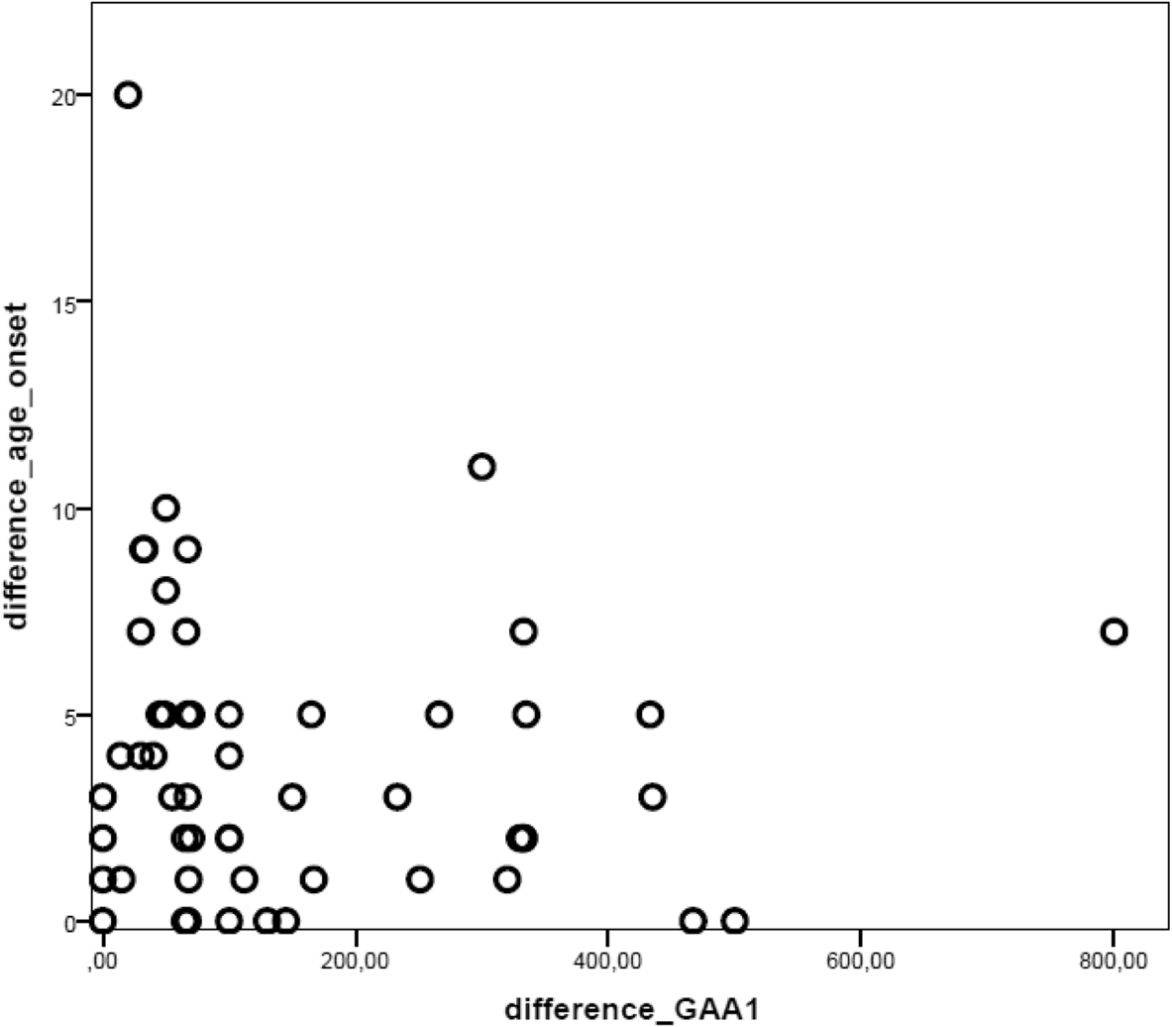
Relationship between age at onset and GAA1-repeat length in FRDA siblings. In 54 siblings’ pairs with FRDA, age at onset and GAA1-repeat length varied between the first sibling to become symptomatic and the latter one. In the graphic, difference in age at onset (in years) across the sibling pairs is plotted against difference in GAA1-repeat length. No correlation was found between the two variables (r = − 0,14, *p* = 0,3)

Considering the categories neurological and non-neurological presentation, 10 out of 54 sib pairs (18.5%) had a discordant onset. Furthermore, marked variability concerning age at onset as well as GAA1 repeat length was observed between siblings (median differences were 3(IQR = 1–5) years and 70(IQR = 48–255) repeats respectively). Difference in age at onset did not correlate with difference in GAA1 repeat length within the sib pairs (r = − 0,14, *p* = 0,3, see also Fig. 3).

As previously mentioned, in eight additional patients pre-symptomatic genetic testing was carried out because of a positive family history. Genetic confirmation was achieved 2.5 ± 2 years before the first symptoms, which then appeared at the mean age of 12 ± 5 years. In two patients out of eight, an isolated non-neurological presentation was reported (cardiomyopathy and diabetes mellitus respectively).

## Discussion

In the present work, we addressed the issue of diagnostic delay in FRDA for the first time. The median time to diagnosis was 3(IQR:1–7) years in this large European cohort, comprehending a mixed population of patients with onset before and after discovery of *FXN* mutations. This value resembles data from general surveys in the field of rare diseases [13], but is somewhat disappointing considering that FRDA is a “common rare disease” among Caucasians. Though, an in-depth look in the EFACTS registry reveals that marked variabilities concerning time to diagnosis are recorded within the FRDA collective.

First of all, the present data highlights the crucial contribution of the discovery of the GAA expansions in *FXN* gene to FRDA diagnosis. Indeed, in the whole cohort, as well in subgroup comparisons, a shorter time to diagnosis is invariably documented when disease presented after 1996. In particular, the increasing availability of genetic testing broke the paradigm of FRDA as a disease of the adolescence, enabling the diagnosis of FRDA with onset up to the mid-70s [18], as evident in our cohort (see Fig. 1).

Despite substantial improvement in the diagnostic process, specific onset features still set a relevant challenge for the clinician and cause significant delay in the diagnosis of FRDA. Indeed, also in the era of genetic diagnosis 1) inaugural non-neurological symptoms and 2) adult onset symptoms go along with a significant diagnostic delay compared to the classical phenotype characterized by onset with gait unsteadiness around puberty. There are a number of reports of disease presentation with isolated cardiomyopathy or scoliosis [2, 15, 16]. The present data shows instead that isolated non-neurological onset is not rare, accounting for ~ 10% of cases. Interestingly, two out of eight patients with pre-symptomatic diagnosis presented with isolated non-neurological symptoms. This suggest that, when transition to manifest disease is closely monitored, non-neurological features may precede clinical ataxia in an even higher percentage of cases. Of note, the EFACTS consortium consists mainly of adult neurological clinics and pediatric cases are underrepresented. Prevalence of non-neurological onset is likely to be higher in pediatric collectives.

Suspecting FRDA in the setting of isolated scoliosis is not reasonable given the prevalence of 1–2% among schoolchildren up to 15 years of age [22]. On the contrary, isolated left ventricular hypertrophy in children is not a common finding. Therefore, testing for FRDA should be considered in individuals diagnosed with this finding [23].

Concerning late-onset FRDA, our results point out that awareness of an adult presentation is still limited. As highlighted by many reviews on the topic [24–26], FRDA testing should be always considered in the first line work-up in adult-onset ataxia after exclusion of secondary causes and in absence of an evident autosomal dominant inheritance.

Measuring the time to diagnosis in rare disease can be challenging, as the disease onset itself can be difficult to define. In the EFACTS registry, age and symptoms at onset are collected retrospectively and based on medical history. Collected information is subject to a recall bias and subclinical neurological signs may be missed. Though, in our experience, age at onset is often re-dated by patients or their parents at time of inclusion as, after becoming aware of FRDA, they can recall earlier abnormalities.

All clinical studies on FRDA consistently reported a significant correlation between the length of the shorter GAA repeat and age at onset. Interestingly in the siblings’ analysis difference in age at onset was not correlated with GAA repeats. This finding may reflect an observational and/or recall bias regarding the age at onset, since the siblings of a newly diagnosed patient are prone to be under higher awareness. More importantly, no correlation between GAA repeat length and age at onset was detected in the group with non-neurological onset. These findings contradict the established correlation between GAA repeat length and disease milestones and suggest that these associations may be valid only when neurological symptoms are considered. Interestingly, cumulative evidence showed that GAA repeat expansions are subjected to somatic instability with accumulation of further contractions/expansions, which can lead to marked differences in the affected tissue and possibly explain lack of correlation between the course non-neurological symptoms and GAA1-repeat length [27]. Eventually, we cannot exclude that the smaller number cases in the siblings’ group and in group with non-neurological onset contributed to the lack of significant correlation between age at onset and GAA1-repeat length.

According to a currently leading hypothesis, FRDA neuropathology has considerable developmental aspects [28]. Indeed, cumulative evidence shows that hypoplasia of the dorsal root ganglia and spinal cord is the dominant neuropathological feature, thus suggesting that anatomical changes predate birth and clinical symptoms [28, 29]. It is not known which factors trigger disease progression and ultimately lead to overt clinical manifestations. Identifying and monitoring paucisymptomatic subjects, without overt ataxia, may offer a unique opportunity to address unanswered issues about the evolution of the disease.

In rare disorders, the interval elapsing between the moment of symptom awareness and definite diagnosis has been described as an “odyssey” [13], during which patients may not receive appropriate management and counseling. Misdiagnosis may also lead to over-testing and inappropriate interventions. In the worst-case scenario, the delay in diagnosis implies also a delay in the access to an available effective therapy. Increasing awareness about FRDA and its presentation represents the first step to address the issue of diagnostic delay. A timely diagnosis accelerates in turn the referral to specialized centers and may pave the way for prompt access to therapies in the future [30].

## Conclusions

We addressed for the first time the issue of diagnostic delay in FRDA in a large multicenter cohort. Our study shows that, even after the introduction of genetic testing 1996, inaugural non-neurological symptoms or adult onset still cause a delay in clinical diagnosis of FRDA. Furthermore, our data highlights that well-known correlations between GAA-repeats and clinical milestones are not valid in the setting of an atypical presentation. The present work adds to the awareness of FRDA in adult patients and in patients with extraordinary symptoms at disease onset. Increasing awareness accelerates diagnosis and may pave the way for a prompt access to therapies in the future.

## Data Availability

The dataset used during the current study can be requested from the EFACTS steering committee.

## Abbreviations

ANCOVA: Analysis of covariance
EFACTS: European Friedreich’s Ataxia Consortium for Translational Studies
FRDA: Friedreich’s Ataxia
*FXN*: Frataxin
IQR: Interquartile range

## References

[1] Vankan P (2013). Prevalence gradients of Friedreich's ataxia and R1b haplotype in Europe co-localize, suggesting a common Palaeolithic origin in the Franco-Cantabrian ice age refuge. J Neurochem.

[2] Parkinson MH, Boesch S, Nachbauer W, Mariotti C, Giunti P (2013). Clinical features of Friedreich’s ataxia: classical and atypical phenotypes. J Neurochem.

[3] Caruso G, Santoro L, Perretti A, Massini R, Pelosi L, Crisci C (1987). Friedreich’s ataxia: electrophysiologic and histologic findings in patients and relatives. Muscle Nerve.

[4] Cossee M, Durr A, Schmitt M, Dahl N, Trouillas P, Allinson P (1999). Friedreich’s ataxia: point mutations and clinical presentation of compound heterozygotes. Ann Neurol.

[5] Filla A, DeMichele G, Caruso G, Marconi R, Campanella G (1990). Genetic data and natural history of Friedreich’s disease: a study of 80 Italian patients. J Neurol.

[6] Durr A, Cossee M, Agid Y, Campuzano V, Mignard C, Penet C (1996). Clinical and genetic abnormalities in patients with Friedreich’s ataxia. N Engl J Med.

[7] Reetz K, Dogan I, Hohenfeld C, Didszun C, Giunti P, Mariotti C (2018). Nonataxia symptoms in Friedreich Ataxia: report from the registry of the European Friedreich’s Ataxia consortium for translational studies (EFACTS). Neurology.

[8] Campuzano V, Montermini L, Molto MD, Pianese L, Cossee M, Cavalcanti F (1996). Friedreich’s ataxia: autosomal recessive disease caused by an intronic GAA triplet repeat expansion. Science..

[9] Galea CA, Huq A, Lockhart PJ, Tai G, Corben LA, Yiu EM (2016). Compound heterozygous FXN mutations and clinical outcome in friedreich ataxia. Ann Neurol.

[10] Reetz K, Dogan I, Costa AS, Dafotakis M, Fedosov K, Giunti P (2015). Biological and clinical characteristics of the European Friedreich’s Ataxia consortium for translational studies (EFACTS) cohort: a cross-sectional analysis of baseline data. Lancet Neurol.

[11] Reetz K, Dogan I, Hilgers RD, Giunti P, Mariotti C, Durr A (2016). Progression characteristics of the European Friedreich’s Ataxia consortium for translational studies (EFACTS): a 2 year cohort study. Lancet Neurol.

[12] Survey of the delay in diagnosis for 8 rare diseases in Europe (‘EurordisCare2’) 2007. Available from: https://www.eurordis.org/publication/survey-delay-diagnosis-8-rare-diseases-europe-%E2%80%98eurordiscare2%E2%80%99.

[13] Ending the diagnostic odyssey for children with a rare disease. Global commission year one report 2019. Available from: https://www.globalrarediseasecommission.com/Report/.

[14] Schulz JB, Boesch S, Burk K, Durr A, Giunti P, Mariotti C (2009). Diagnosis and treatment of Friedreich ataxia: a European perspective. Nat Rev Neurol.

[15] Quercia N, Somers GR, Halliday W, Kantor PF, Banwell B, Yoon G (2010). Friedreich ataxia presenting as sudden cardiac death in childhood: clinical, genetic and pathological correlation, with implications for genetic testing and counselling. Neuromuscul Disord.

[16] Leonard H, Forsyth R (2001). Friedreich’s ataxia presenting after cardiac transplantation. Arch Dis Child.

[17] Bhidayasiri R, Perlman SL, Pulst SM, Geschwind DH (2005). Late-onset Friedreich ataxia: phenotypic analysis, magnetic resonance imaging findings, and review of the literature. Arch Neurol.

[18] Lecocq C, Charles P, Azulay JP, Meissner W, Rai M, N'Guyen K (2016). Delayed-onset Friedreich’s ataxia revisited. Mov Disord.

[19] Berciano J, Mateo I, De Pablos C, Polo JM, Combarros O (2002). Friedreich ataxia with minimal GAA expansion presenting as adult-onset spastic ataxia. J Neurol Sci.

[20] Coppola G, De Michele G, Cavalcanti F, Pianese L, Perretti A, Santoro L (1999). Why do some Friedreich’s ataxia patients retain tendon reflexes? A clinical, neurophysiological and molecular study. J Neurol.

[21] Miller GA, Chapman JP (2001). Misunderstanding analysis of covariance. J Abnorm Psychol.

[22] Konieczny MR, Senyurt H, Krauspe R (2013). Epidemiology of adolescent idiopathic scoliosis. J Child Orthop.

[23] Elliott PM, Anastasakis A, Borger MA, Borggrefe M, Cecchi F, Authors/Task Force m (2014). 2014 ESC guidelines on diagnosis and management of hypertrophic cardiomyopathy: the Task Force for the diagnosis and Management of Hypertrophic Cardiomyopathy of the European Society of Cardiology (ESC). Eur Heart J.

[24] Fogel BL, Perlman S (2007). Clinical features and molecular genetics of autosomal recessive cerebellar ataxias. Lancet Neurol.

[25] Renaud M, Tranchant C, Martin JVT, Mochel F, Synofzik M, van de Warrenburg B (2017). A recessive ataxia diagnosis algorithm for the next generation sequencing era. Ann Neurol.

[26] Synofzik M, Nemeth AH (2018). Recessive ataxias. Handb Clin Neurol.

[27] Long A, Napierala JS, Polak U, Hauser L, Koeppen AH, Lynch DR (2017). Somatic instability of the expanded GAA repeats in Friedreich’s ataxia. PLoS One.

[28] Koeppen AH, Becker AB, Qian J, Gelman BB, Mazurkiewicz JE (2017). Friedreich Ataxia: developmental failure of the dorsal root entry zone. J Neuropathol Exp Neurol.

[29] Koeppen AH, Becker AB, Qian J, Feustel PJ (2017). Friedreich Ataxia: hypoplasia of spinal cord and dorsal root ganglia. J Neuropathol Exp Neurol.

[30] Reata Announces Positive Topline Results from the MOXIe Registrational Trial of Omaveloxolone in Patients with Friedreich’s Ataxia 2019. Available from: https://www.reatapharma.com/press-releases/reata-announces-positive-topline-results-from-the-moxie-registrational-trial-of-omaveloxolone-in-patients-with-friedreichs-ataxia/.

